# Transcriptome analysis of *Sporisorium scitamineum* reveals critical environmental signals for fungal sexual mating and filamentous growth

**DOI:** 10.1186/s12864-016-2691-5

**Published:** 2016-05-16

**Authors:** Meixin Yan, Weijun Dai, Enping Cai, Yi Zhen Deng, Changqing Chang, Zide Jiang, Lian-Hui Zhang

**Affiliations:** Guangdong Province Key Laboratory of Microbial Signals and Disease Control, College of Agriculture, South China Agricultural University, Guangzhou, Peoples’ Republic of China; Guangdong Innovative and Entepreneurial Research Team of Sociomicrobiology Basic Science and Frontier Technology, South China Agricultural University, Guangzhou, Peoples’ Republic of China; Integrative Microbiology Research Centre, South China Agricultural University, Guangzhou, Peoples’ Republic of China

**Keywords:** Mating, Sugarcane smut, bE/bW heterodimeric transcriptional factor, Glucose, Hog1

## Abstract

**Background:**

*Sporisorium scitamineum* causes the sugarcane smut disease, one of the most serious constraints to global sugarcane production. *S. scitamineum* possesses a sexual mating system composed of two mating-type loci, *a* and *b* locus. We previously identified and deleted the *b* locus in *S. scitamineum*, and found that the resultant *SsΔMAT-1b* mutant was defective in mating and pathogenicity.

**Results:**

To further understand the function of b-mating locus, we carried out transcriptome analysis by comparing the transcripts of the mutant strain *SsΔMAT-1b*, from which the SsbE1 and SsbW1 homeodomain transcription factors have previously been deleted, with those from the wild-type *MAT-1* strain. Also the transcripts from *SsΔMAT-1b* X *MAT-2* were compared with those from wild-type *MAT-1* X *MAT-2* mating. A total of 209 genes were up-regulated (*p* < 0.05) in the *SsΔMAT-1b* mutant, compared to the wild-type *MAT-1* strain, while 148 genes down-regulated (*p* < 0.05). In the mixture, 120 genes were up-regulated (*p* < 0.05) in *SsΔMAT-1b* X *MAT-2*, which failed to mate, compared to the wild-type *MAT-1* X *MAT-2* mating, and 271 genes down-regulated (*p* < 0.05). By comparing the up- and down-regulated genes in these two sets, it was found that 15 up-regulated and 37 down-regulated genes were common in non-mating haploid and mating mixture, which indeed could be genes regulated by b-locus. Furthermore, GO and KEGG enrichment analysis suggested that carbon metabolism pathway and stress response mediated by Hog1 MAPK signaling pathway were altered in the non-mating sets.

**Conclusions:**

Experimental validation results indicate that the bE/bW heterodimeric transcriptional factor, encoded by the *b*-locus, could regulate *S. scitamineum* sexual mating and/or filamentous growth via modulating glucose metabolism and Hog1-mediating oxidative response.

**Electronic supplementary material:**

The online version of this article (doi:10.1186/s12864-016-2691-5) contains supplementary material, which is available to authorized users.

## Background

Sugarcane smut is a devastating disease in sugarcane growing areas globally. The characteristic symptom of the disease is a black or gray growth that is referred to as a “smut whip” [1]. Sugarcane smut is caused by the fungus *S. scitamineum*, a bipolar species [2, 3] with two mating type strains *MAT-1* and *MAT-2* [4] producing haploid sporidia by budding. The compatible sporidia fuse to develop pathogenic dikaryotic hyphae, which grow within the stalk of sugarcane and form diploid teliospores to complete the pathogenic life cycle [3]. The teliospores are disseminated by wind or rain splashes and germinate to form four sporidia, and initiate next round of life cycle by mating. The sexual mating process of *S. scitamineum* is similar to the maize pathogen *Ustilago maydis*, which is regulated by two unlinked mating type loci, *a* locus and *b* locus [5–7]. The bi-allelic *a* loci that encode a pheromone/pheromone receptor system that is responsible for recognition of the opposite haploid sporidia and formation of conjugation tubes [8]. The *b* locus composed of the *bE* and *bW* genes, encoding a heterodimeric transcription factor to maintain the dikaryotic filament and promote subsequent penetration of the host plant, after fusion of the sporidia [8–10].

It has been reported that in *U. maydis*, the bE/bW transcription factor acts through a regulatory cascade to affect various pathways in triggering pathogenic development, including cell cycle regulation, mitosis and DNA replication [11]. However, the physiology of *S. scitamineum* mating is largely unknown, due to unavailability of genome sequence and effective method of genetic manipulation, previously. Recently, with the genome sequencing performed by Que et al. [2] and Taniguti et al. [12], and optimizing of the ATMT transformation procedure for *S. scitamineum* [13], investigation on *S. scitamineum* differentiation and pathogenesis on molecular level becomes feasible. Recently, we identified and characterized a b-locus homolog in *S. scitamineum*, and found that it is essential for sexual mating and filamentous growth [14], but the underlying mechanism remained unclear. Given that b-locus encodes a homeodomain transcription complex, comparative transcriptome analysis may provide useful clues to possible b-locus target gene(s) and functional study of such candidate gene(s) may reveal the molecular basis of b-locus regulating *S. scitamineum* sexual mating and/or filamentous growth. Therefore, we carried out transcriptome analysis with wild-type *MAT-1* and *SsΔMAT-1b* mutant, and with mating and non-mating mixtures of *S. scitamineum* haploids. Our study identified several potential target genes of b-locus encoding transcriptional factor, that are likely involved in *S. scitamineum* sexual mating and/or filamentous growth, and further reveals two critical endogenous/environmental cues: nutrient and redox homeostasis, for mating and/or filementous growth in *S. scitamineum*.

## Methods

### Growth conditions and strains used in this study

Teliospores of sugarcane smut were collected from the fields in Guangdong province of China (21°12′ 36′′ N; 101°10′ 12′′ E), and no specific permissions were required for sampling diseased plants in this location. Haploid colonies of *MAT-1* and *MAT-2* were isolated from these teliospores by serial dilution and plating on YePSA medium, as previously described [15]. Synthetic complete dextrose (SCD) medium is consisted of 0.7 % (wt/vol) yeast nitrogen base without amino acids, 0.17 % complete amino acids powder, and 2 % (wt/vol) glucose [16]. Synthetic complete (SC) medium was formulated as SCD medium without addition of glucose [16].

### RNA extraction and sequencing strategies

TRIzol Reagent (Life Technologies, UK) was used for Total RNA extraction from haploid *MAT-1* and *SsΔMAT-1b* mutant. *MAT-1* and *MAT-2* haploids were mixed and plated on YePSA medium for 24 h before total RNA extraction with TRIzol Reagent. Similarly, *SsΔMAT-1b* and *MAT-2* haploids were mixed and inoculated on YePSA medium for 24 h before total RNA extraction.

Libraries were constructed following Illumina manufacturer’s protocol of the “TruSeq RNA Sample Prep v2 Low Throughput (LT)” kit. Paired-end sequencing was performed on the Illumina HiSeq™2000. Reads were analyzed by FASTQC (http://www.bioinformatics.babraham.ac.uk/projects/fastqc/) and low quality bases (phred ≥20), Illumina adapters and poly-A tails were removed using the NGS QC Toolkit v2.3.3 (http://59.163.192.90:8080/ngsqctoolkit/) [17].

### Transcriptome assembly and annotations

*De novo* short read assembly was performed using tophat and cufflinks softwares [18]. The assembled reads were mapped to the complete genome of *S. scitamineum* SSC39B strain (ftp://ftp.ncbi.nlm.nih.gov/genomes/genbank/fungi/Sporisorium_scitamineum/latest_assembly_versions/GCA_000772675.1_Sporisorium_scitamineum_v1) using Tophat and Bowtie2 [19].

Unigene generated by *De novo* short read assembly was aligned to NCBI NR Database (ftp://ftp.ncbi.nih.gov/blast/db), SWISSPROT Database (http://www.uniprot.org/downloads), and KOG Database (Clusters of orthologous groups for eukaryotic complete genomes, ftp://ftp.ncbi.nih.gov/pub/COG/KOG/kyva), respectively. Unigene encoding proteins with high similarity (e <1e-5) to the known proteins in aforementioned databases were used to annotate the corresponding Unigene. GO annotation was performed by Blast2GO software [20] and the database http://www.geneontology.org/. KEGG annotation was performed with the database http://www.genome.jp/kegg/pathway.html [21].

### Transcriptome analysis

Differential transcript accumulation among treatments (*SsΔMAT-1b* vs *MAT1*, *SsΔMAT-1b* X *MAT-2* vs *MAT-1* X *MAT-2*) was observed using bowtie2 (http://bowtie-bio.sourceforge. net/bowtie2/manual.shtml) [19] and eXpress [22]. The gene expression level is calculated by using FPKM method (fragments Per kb per Million reads) [22]. Baggerley’s test and the false discovery rate (FDR) with a significance level of ≤0.05 and the absolute value of Log2Ratio ≥1 was set as the threshold to judge the significance of gene expression difference.

GO enrichment analysis was performed as firstly mapping all DEGs (Differential Expressed Genes) to GO terms in the database (http://www.geneontology.org/), calculating gene numbers for every term, then using hypergeometric test to find significantly enriched GO terms in the input list of DEGs, based on GO::TermFinder (http://smd.stanford.edu/help/GOTermFinder/GO_ TermFinder_help.shtml/). *P* value was calculated using the following formula:

$$ P=1-{\displaystyle \sum_{i=0}^{m-1}\frac{\left(\begin{array}{c}\hfill M\hfill \\ {}\hfill i\hfill \end{array}\right)\left(\begin{array}{c}\hfill N-M\hfill \\ {}\hfill n-i\hfill \end{array}\right)}{\left(\begin{array}{c}\hfill N\hfill \\ {}\hfill n\hfill \end{array}\right)}} $$

Where N is the number of all genes with GO annotation; n is the number of DEGs in N; M is the number of all genes that are annotated to certain GO terms; m is the number of DEGs in M. The calculated p-value goes through Bonferroni Correction [23], taking corrected *p*-value ≤ 0.05 as a threshold. GO score was calculated as follows: $$ Enrichmentscore=\raisebox{1ex}{$\frac{m}{n}$}\!\left/ \!\raisebox{-1ex}{$\frac{M}{N}$}\right. $$.

KEGG database is used to perform pathway enrichment analysis of DEGs. The calculating formula is the same as that in GO analysis. Here N is the number of all genes that with KEGG annotation, n is the number of DEGs in N, M is the number of all genes annotated to specific pathways, and m is the number of DEGs in M.

## Results

### Unigenes identification and gene annotation

**Fig. 1:**
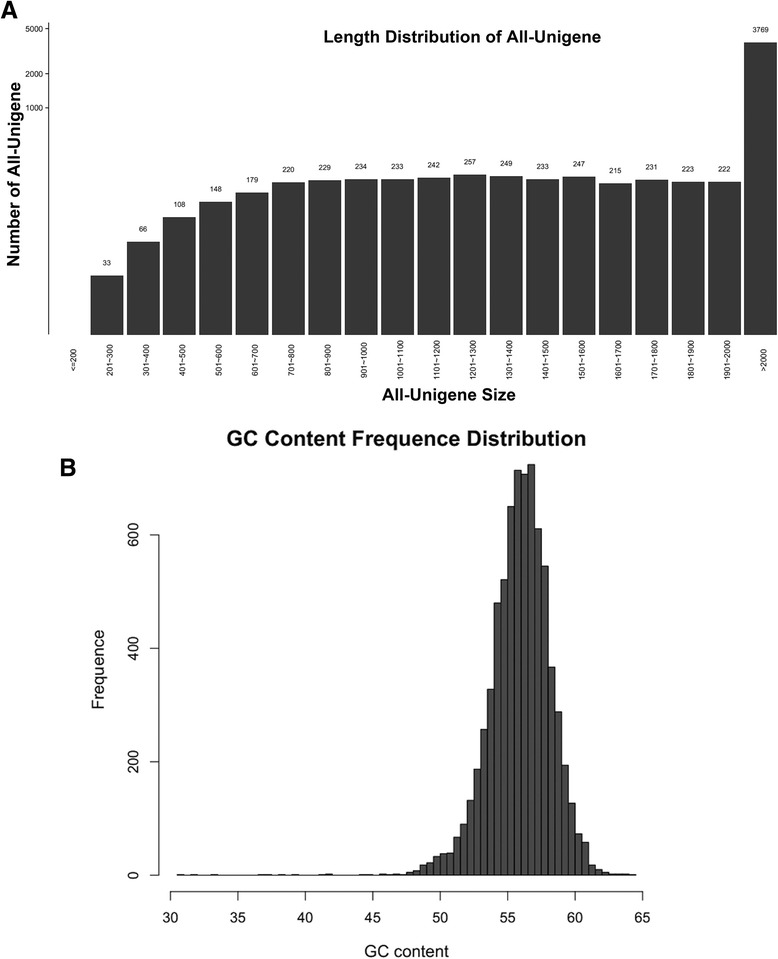
Length and GC-content of All-Unigene. **a** Bar chart depicting length distribution of All-Unigene identified in this study. **b** GC content frequency distribution of All-Unigene of this study

Our RNAseq analysis produced a total length of 17.8344 Mb (Table 1) for all the transcripts, out of 2G clean sequencing data, representing about 100 X coverage of the transcriptome. Compared to previous published genomic sequence of *S. scitamineum* [2, 12], the total length of sequence is slightly low, likely due to the fact that only transcripts (with poly-A tails) were anchored and sequenced in this study. *De novo* assembly of transcripts was performed as described in Methods. We identified 7341 unigenes in total, with length from 145 bp to 16628 bp (Table 1). Most of the identified unigenes are of 200–2000 bp (Fig. 1a), and GC content is within the range of 50–60 % (Fig. 1b). The unigenes were mapped to NR, SWISSPROT, and KOG Database for annotation, as listed in Additional file 1: Table S1.

**Table 1:** Unigene statistics

	All	> = 200 bp	> = 500 bp	> = 1000 bp	Total Length (Mb)	Max Length	Min Length	Avg Length
PRJNA240344	-	-	-	-	19.7235	-	-	-
PRJEB5169	7711	-	-	-	19.4279	-	-	-
PRJNA275631	6677	-	-	-	20.0676	-	-	-
Unigene	7341	7338	7131	6123	17.8344	16628	145	2429.42

### Transcriptome analysis identified differentially expressed genes between mating and non-mating strains/conditions

In this study, we compared two sets of non-mating vs mating strain/condition, with an aim to identify the genes related to *S. scitamineum* mating and likely regulated by *b*-locus. Differentially Expressed Genes (DEGs) were identified in the *SsΔMAT-1b* mutant compared to the wild-type *MAT-1* strain, as well as in the non-mating mixture, *SsΔMAT-1b X MAT-2*, compared to the mating mixture of *MAT-1* X *MAT-2*. DEGs of significance (*p* ≤ 0.05) in the haploid and mating set were listed in Additional file 2: Table S2 and Additional file 3: Table S3 respectively. In total, there are 357 DEGs identified in the *SsΔMAT-1b* mutant, among which 209 genes were up-regulated and 148 down-regulated (Table 2). Under mating condition, a total of 391 genes were differentially expressed in the non-mating mixture, with 120 up-regulated and 271 down-regulated (Table 2). By comparing the up- and down-regulated genes in these two conditions, we found that 15 up-regulated and 37 down-regulated genes were common in non-mating haploid and mating mixture. We listed in Table 3 for those with annotation in SWISSPROT Database.

**Table 2:** DEGs statistics

Control	Case	Up_diff	Down_diff	Total_diff
*MAT-1*	*b*-deletion	209	148	357
*MAT-1 b*-deletion + *MAT-2*	*MAT-1* + *MAT-2*	120	271	391

**Table 3:** List of Up- and Down- regulated genes common in *SsΔMAT-1b* vs wild-type *MAT-1* and *SsΔMAT-1b X MAT-2* vs wild-type *MAT-1 X MAT-2* comparing sets

Swiss ID	Length (bp)	Fold change	*p* value	Fold change	*p* value	swiss.Description
		*SsΔMAT-1b vs MAT-1*		*SsΔMAT-1b* X *MAT-2 vs MAT-1* X *MAT-2*		
sp|P38938|CEK1_SCHPO	13208	13.42056	1.68E-06	3.44586	0.01867	Serine/threonine-protein kinase cek1 OS = Schizosaccharomyces pombe (strain 972/ATCC 24843) GN = cek1 PE = 1 SV = 3
sp|P39960|BEM2_YEAST	8330	1.796835	0.023621	1.718937	0.023855	GTPase-activating protein BEM2/IPL2 OS = Saccharomyces cerevisiae (strain ATCC 204508/S288c) GN = BEM2 PE = 1 SV = 1
sp|Q6R3K9|YSL2_ARATH	2834	1.946333	0.003032	1.913564	0.010979	Metal-nicotianamine transporter YSL2 OS = Arabidopsis thaliana GN = YSL2 PE = 2 SV = 1
sp|Q9JME5|AP3B2_MOUSE	9407	12.37208	9.87E-06	4.222222	2.16E-05	AP-3 complex subunit beta-2 OS = Mus musculus GN = Ap3b2 PE = 1 SV = 2
sp|Q12019|MDN1_YEAST	16628	1.87115	0.005704	2.001882	0.002886	Midasin OS = Saccharomyces cerevisiae (strain ATCC 204508/S288c) GN = MDN1 PE = 1 SV = 1
sp|Q54YH4|DHKB_DICDI	6828	Inf	0.007605	10.75	0.005969	Hybrid signal transduction histidine kinase B OS = Dictyostelium discoideum GN = dhkB PE = 1 SV = 1
sp|A2BGA0|RFX4_DANRE	4929	6.911589	1.76E-06	1.937224	0.015736	Transcription factor RFX4 OS = Danio rerio GN = rfx4 PE = 2 SV = 1
sp|Q4P3W3|DBP10_USTMA	3384	12.41402	0.025351	4.851852	0.046387	ATP-dependent RNA helicase DBP10 OS = Ustilago maydis (strain 521/FGSC 9021) GN = DBP10 PE = 3 SV = 1
sp|P56584|SID1_USTMA	2399	1.764062	0.017173	2.524876	0.016416	L-ornithine 5-monooxygenase OS = Ustilago maydis (strain 521/FGSC 9021) GN = SID1 PE = 2 SV = 2
sp|P36619|PMD1_SCHPO	7057	2.328467	0.01718	2.990596	0.006957	Leptomycin B resistance protein pmd1 OS = Schizosaccharomyces pombe (strain 972/ATCC 24843) GN = pmd1 PE = 3 SV = 2
sp|Q4PC06|HOG1_USTMA	5538	3.954272	0.001332	2.294118	0.037876	Mitogen-activated protein kinase HOG1 OS = Ustilago maydis (strain 521/FGSC 9021) GN = HOG1 PE = 3 SV = 1
sp|P0CJ65|ATB54_ARATH	2265	0.02171	6.20E-19	0.178523	3.40E-06	Homeobox-leucine zipper protein ATHB-54 OS = Arabidopsis thaliana GN = ATHB-54 PE = 2 SV = 1
sp|P22943|HSP12_YEAST	1053	0.168388	9.19E-15	0.552964	0.01578	12 kDa heat shock protein OS = Saccharomyces cerevisiae (strain ATCC 204508/S288c) GN = HSP12 PE = 1 SV = 1
sp|O14094|PPX1_SCHPO	2702	0.36517	0.002301	0.144718	0.000202	Putative exopolyphosphatase OS = Schizosaccharomyces pombe (strain 972/ATCC 24843) GN = SPAC2F3.11 PE = 3 SV = 1
sp|Q6CHP9|CCM1_YARLI	2835	0.451759	0.010845	0.049724	1.68E-07	Mitochondrial group I intron splicing factor CCM1 OS = Yarrowia lipolytica (strain CLIB 122/E 150) GN = CCM1 PE = 3 SV = 1
sp|P22018|B4_USTMD	1539	0	9.33E-23	0.325834	0.001866	Mating-type locus allele B4 protein OS = Ustilago maydis PE = 3 SV = 1
sp|Q8VZ80|PLT5_ARATH	2427	0.452944	0.022087	0.493914	0.003544	Polyol transporter 5 OS = Arabidopsis thaliana GN = PLT5 PE = 1 SV = 2
sp|Q4WFX9|LAP2_ASPFU	2169	0.053682	0.00954	0.337114	4.19E-05	Probable leucine aminopeptidase 2 OS = Neosartorya fumigata (strain ATCC MYA-4609/Af293/CBS 101355/FGSC A1100) GN = lap2 PE = 3 SV = 2
sp|Q5UP73|YR614_MIMIV	1884	0.353454	2.79E-06	0.340428	1.15E-05	Putative band 7 family protein R614 OS = Acanthamoeba polyphaga mimivirus GN = MIMI_R614 PE = 3 SV = 1
sp|Q8K4J6|MKL1_MOUSE	736	0.600119	0.019664	0.330712	5.01E-06	MKL/myocardin-like protein 1 OS = Mus musculus GN = Mkl1 PE = 1 SV = 2
sp|Q9RPT1|RHLG_PSEAE	1073	0.337766	0.035915	0.16568	0.019132	Rhamnolipids biosynthesis 3-oxoacyl-[acyl-carrier-protein] reductase OS = Pseudomonas aeruginosa (strain ATCC 15692/PAO1/1C/PRS 101/LMG 12228) GN = rhlG PE = 1 SV = 1
sp|P80299|HYES_RAT	1160	0.467807	0.005301	0.425344	0.024971	Bifunctional epoxide hydrolase 2 OS = Rattus norvegicus GN = Ephx2 PE = 1 SV = 1
sp|P36914|AMYG_ASPOR	2852	0.569668	0.010942	0.419058	0.010065	Glucoamylase OS = Aspergillus oryzae (strain ATCC 42149/RIB 40) GN = glaA PE = 2 SV = 2
sp|P34211|YUAR_ECOLI	2743	0.226761	3.58E-06	0.318024	3.27E-05	Putative hydrolase YuaR OS = Escherichia coli (strain K12) GN = yuaR PE = 3 SV = 3
sp|O35750|SHOX2_RAT	3616	0.490521	0.005405	0.55	0.023074	Short stature homeobox protein 2 (Fragment) OS = Rattus norvegicus GN = Shox2 PE = 2 SV = 2
sp|Q767C8|IH5GT_IRIHO	2482	0.263235	3.53E-09	0.630828	0.048192	Cyanidin 3-O-rutinoside 5-O-glucosyltransferase OS = Iris hollandica GN = 5GT PE = 1 SV = 1
sp|O42922|YBIH_SCHPO	4584	0.240982	5.71E-10	0.61912	0.037253	Uncharacterized MFS-type transporter C16A3.17c OS = Schizosaccharomyces pombe (strain 972 / ATCC 24843) GN = SPBC16A3.17c PE = 3 SV = 1

Among the 12 up-regulated and 16 down-regulated genes listed in Table 3, we noticed that genes encoding components of signaling pathway, e.g. MAPK Cek1 (involved in mitosis in yeast [24] and fungicidal activity in *Candida albicans* [25]) and Hog1 (oxidative or osmotic stress response [26–28]), GTPase-activating protein BEM2/IPL2 (for cellular morphogenesis and interacting with mitosis regulator in yeast [29]), or histidine kinase (possibly involved in two-component signal pathway [30]) were up-regulated with deletion of b-locus. Also, proteins involved vesicular trafficking (AP-3 complex subunit) or metal-nicotianamine transporter YSL2 were possibly repressed by b-locus transcriptional factor (Table 3). Another transcriptional factor, RFX4, and an RNA helicase were potentially repressed by b-locus too (Table 3). This result indicates that b-locus may negatively regulate some signaling pathway and repressed transcription of a set of downstream genes, directly or indirectly, after sexual mating induced and during filamentous growth. On the other hand, genes induced, directly or indirectly, by b-locus include several other transcriptional factors, e.g. ATHB-54 [31], MKL/myocardin-like protein [32], Short stature homeobox protein 2 (Shox2; related to growth and development in human [33]; Table 3). b-locus may also induce regulators involved in biosynthesis, including polyol transporter 5, Rhamnolipids biosynthesis 3-oxoacyl-[acyl-carrier-protein] reductase, MFS-type transporter, and several hydrolases or Glucoamylase, during mating and/or filamentous growth (Table 3). PKA and MAPK signaling pathway were found to be involved in b-locus regulating sexual mating and/or filamentous growth in *U. maydis* [11]. Here in our study, we also identified component of MAPK pathway, Cek1 and Hog1, that was potentially regulated by *S. scitamineum* b-locus, but not among those identified in *U. maydis*. Our finding indicates that *S. scitamineum* b-locus may regulate small molecular (e.g. metal-nicotianamine, polyol) transport, vesicular trafficking, biosynthesis, stress-response mediated by MAPK signaling (Hog1), and a cascade of transcriptional network, during mating and/or filamentous growth. The candidate genes listed in Table 3 are of great interest in our future investigation, in terms of elucidating physiology and molecular mechanism of *S. scitamineum* differentiation and pathogenesis.

### Identification of starch/sucrose metabolism and Hog1 MAPK pathway in fungal mating

**Fig. 2:**
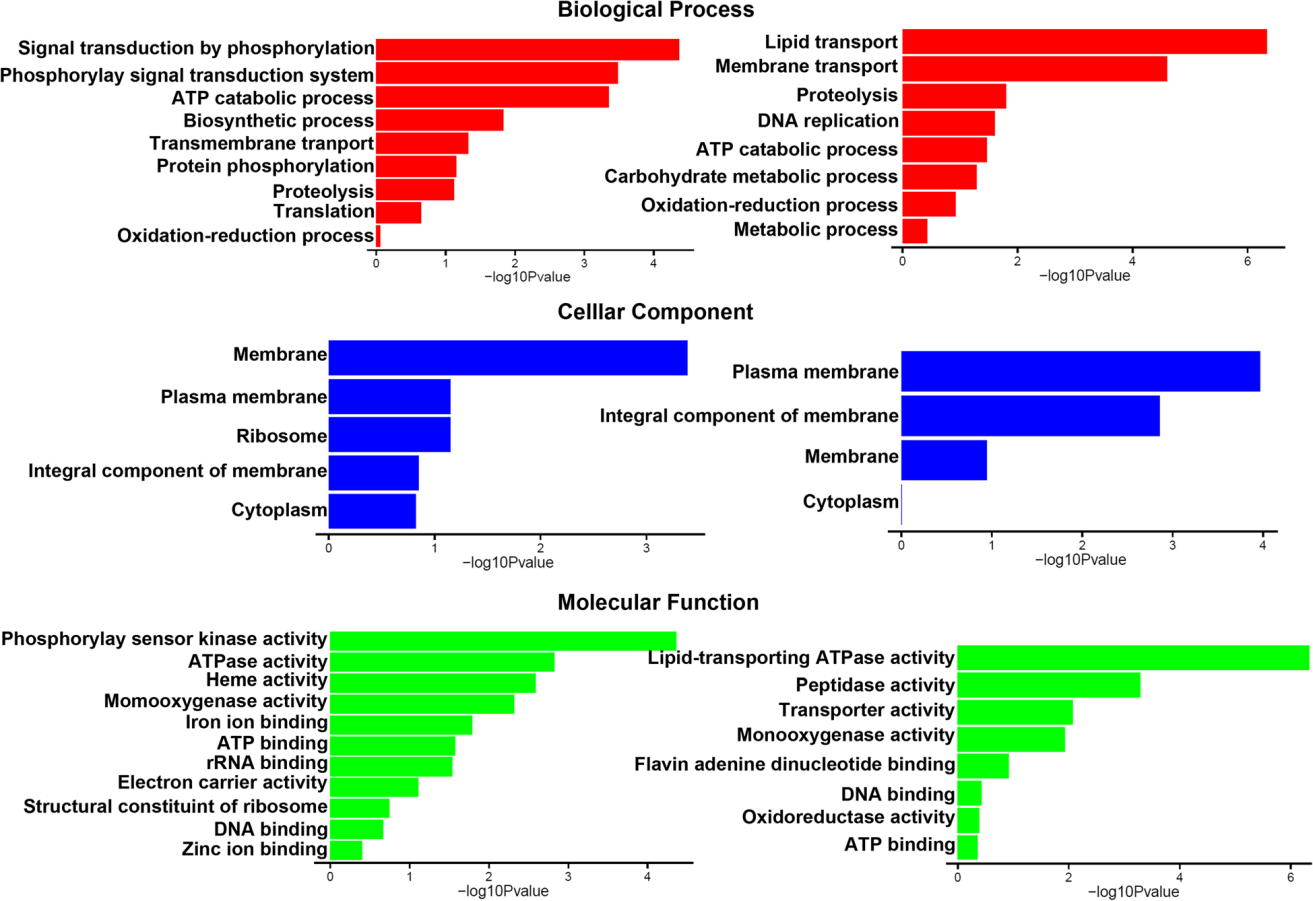
GO-enrichment of DEGs. Left panal: *SsΔMAT-1b* mutant vs wild-type *MAT-1*; right panal: *SsΔMAT-1b* X *MAT-2* mixture vs *MAT-1* X *MAT-2*. GO terms were catalogued as Biological Process, Cellular Component, and Molecular Function

As an international standard gene functional classification system, Gene Ontology (GO), offers a dynamic-updated controlled vocabulary, as well as a strictly defined concept to comprehensively describe properties of genes and their products in any organism [34]. Therefore GO enrichment analysis of the aforementioned DEGs may further reveal the functional relevance of *b*-locus regulating genes and *S. scitamineum* mating. Enriched GO (for both up- and down- regulated) in the haploid and mating sets were listed in Additional file 4: Table S4 and Additional file 5: Table S5 respectively, and schematically represented following three ontologies (molecular function, cellular component and biological process) as in Fig. 2. Among them, we noticed that the genes involved in membrane transport, oxidation-reduction process and ATP-binding were overall differentially regulated in non-mating haploid (*SsΔMAT-1b* mutant), as well as in non-mating mixture (*SsΔMAT-1b X MAT-2*, Fig. 2). However, some particular genes associated with the membrane transport process (GO: 0055085) were up-regulated, while some others, enriched in the same GO term, were down-regulated, in both non-mating haploid and non-mating mixture (Additional file 4: Table S4 and Additional file 5: Table S5). Similar situation occurred for oxidation-reduction process (GO: 0055114; Additional file 4: Table S4 and Additional file 5: Table S5) as well as ATP-binding (GO: 0005524; Additional file 4: Table S4 and Additional file 5: Table S5). On the other hand, ATP catabolic process (GO: 0006200) was up-regulated in both *SsΔMAT-1b* mutant and *SsΔMAT-1b X MAT-2* mixture (Additional file 4: Table S4 and Additional file 5: Table S5), indicating that *S. scitamineum* mating may repress ATP catabolism. In summary, GO terms enrichment analysis further verifies that metabolism, biosynthsis, transmembrane transport and redox homeostasis would be tightly regulated by b-locus during *S. scitamineum* mating and/or filamentous growth.

Genes usually interact with each other to play roles in certain biological functions. Pathway-based analysis helps to further understand the biological functions of unigenes. KEGG-enrichment analysis thus was carried out to identify significantly enriched metabolic pathways or signal transduction pathways in DEGs comparing with the whole genome background [21]. Enriched KEGG terms were listed in Additional file 6: Table S6 and Additional file 7: Table S7, for *SsΔMAT-1b* vs wild-type *MAT-1* and the non-mating mixture of *SsΔMAT-1b* X *MAT-2* vs wild-tyype *MAT-1* X *MAT-2*, respectively. Among the enriched pathways, we observed that starch and sucrose metabolism pathway (ko00500; Additional file 8: Figure S1) was commonly found in both haploid and mating sets. The predicted outcome of differentially regulation of this pathway was that glucose production would be reduced, while accumulation of 1,3-β-glucan would be increased (Additional file 8: Figure S1), in *SsΔMAT-1b* or non-mating mixture. Another commonly up-regulated gene, Hog1 (p38), was also found in enriched KEGG pathway (ko04010, MAPK signaling) in both *SsΔMAT-1b* or non-mating mixture (Additional file 9: Figure S2). Hog1 mediates osmo- and oxidative stress response in yeast and fungi [26–28], and is important for mating capacity in *Candida albicans* [16]. We infer that carbohydrate metabolism as well as redox homeostasis may play important roles in *S. scitamineum* mating, and be subjective to regulation (directly or indirectly) by the *b*-locus.

### Starch/sucrose metabolism and Hog1 MAPK pathway may regulate *S. scitamineum* mating

**Fig. 3:**
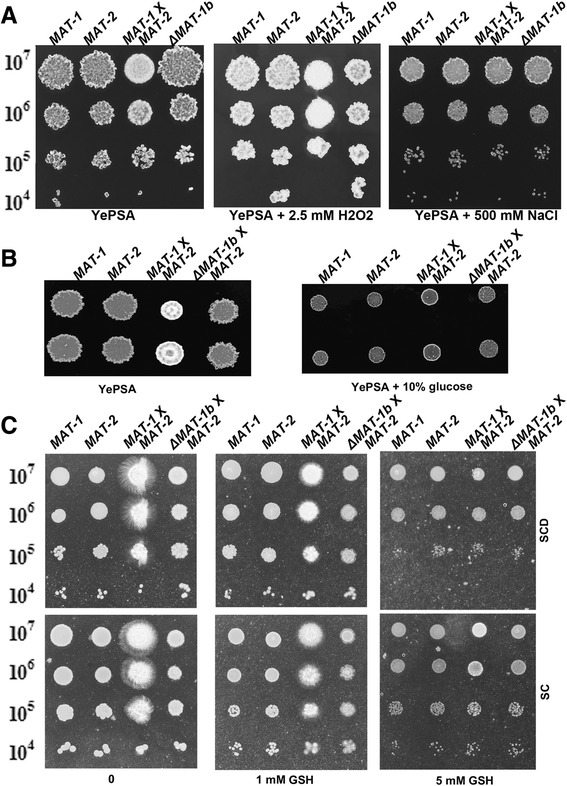
Starch/sucrose metabolism and Hog1 MAPK pathway are likely involved in *S. scitamineum* mating, and subject to regulation of the *b*-locus. **a** Serially diluted cells of *MAT-1*, *MAT-2*, *MAT-1* X *MAT-2*, and *SsΔMAT-1b*, were spotted onto YePSA medium supplemented with 2.5 mM hydrogen peroxide or 500 mM NaCl. **b** Cells of *MAT-1*, *SsΔMAT-1b*, *MAT-1* X *MAT-2*, and *SsΔMAT-1b* X *MAT-2*, were spotted onto YePSA medium with or without 10 % (wt/vol) of glucose. **c** Serially diluted cells of *MAT-1*, *SsΔMAT-1b*, *MAT-1* X *MAT-2*, and *SsΔMAT-1b* X *MAT-2*, were spotted onto SCD or SC medium, with 1 mM or 5 mM GSH

**Fig. 4:**
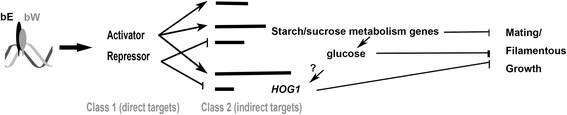
Proposed Model. bE and bW proteins derived from opposite mating type form functional transcriptional complex to activate or repress Class 1 (direct) target genes. Class 2, indirect targets, are in turn activated or repressed by products encoded by Class 1 targets. Starch/sucrose metabolism genes as well as *HOG1* may be indirectly repressed by the bE/bW transcriptional complex, during mating. *b*-repressed glucose metabolism gene may promote production of glucose, which may repress mating and/or filamentous growth. Meanwhile, glucose production may lead to elevated intracellular oxidative level, and thus induce Hog1 MAPK pathway, which also negatively regulates mating and/or filamentous growth. Overall, *b*-locus may act in shutting down mating/filamentous growth inhibitors, including high level of glucose and Hog1 MAPK signaling

To verify the involvement of starch/sucrose metabolism and Hog1 MAPK pathway in *S. scitamineum* mating, we tested the growth of the wild-type *MAT-1*, *MAT-2* and *SsΔMAT-1b* mutant, as well as mating *MAT-1* X *MAT-2* mixtures, under osmotic and oxidative stresses. The results showed that *SsΔMAT-1b* was more resistant to oxidative stress, compared to the wild-type *MAT-1* as well as mating mixture (Fig. 3a middle panel). However, wild-type *MAT-2* also showed higher resistance to H2O2 when cultured alone but not in mating condition (Fig. 3a middle panel). Osmotic stress imposed by 500 mM NaCl repressed the filamentous growth in the mating mixture of *MAT-1* X *MAT-2* (Table 3A right panel). However, the colonial growth was indistinguishable between the wild-type *MAT-1* and *SsΔMAT-1b* mutant strain, under the same osmotic stress (Fig. 3a right panel). On the other hand, the YePSA medium supplemented with high concentration (10 %, wt/vol) of glucose repressed filamentous growth in the mating mixture of *MAT-1* X *MAT-2* (Fig. 3b). In contrast, glucose-depleted medium (SC) was more favorable for filamentous growth in mating mixture of *MAT-1* X *MAT-2*, compared to the SCD medium containing 2 % glucose (Fig. 3c). As 1,3-β-glucan is an effective anti-oxidant, the significant enhancement of Hog1 transcripts in non-mating haploid/mixture may be an indirect consequence of elevated intracellular oxidative level in non-mating *S. scitamineum* haploid and mixture. Furthermore, we tested the effect of anti-oxidant, Glutathione (GSH) on colonial and filamentous growth of haploid and mating strains. All the strains were more resistant to GSH on SC (glucose-deplete) medium compared to SCD (glucose-containing) medium (Fig. 3c). This indicates that the glucose may indeed be utilized for synthesis of anti-oxidant 1,3-β-glucan, therefore depletion of glucose resulted in more resistance to GSH, another anti-oxidant. Overall, these results indicate that glucose may play a negative role in promoting *S. scitamineum* mating and/or filamentous growth, and the *b*-locus encoding heterodimeric transcriptional factor may regulate starch/sucrose metabolism on transcriptional level. We further predicted, based on transcriptome analysis, that the *b*-locus encoded heterodimeric transcriptional factor may regulate *S. scitamineum* mating and/or filamentous growth by promoting synthesis of 1,3-β-glucan (probably from D-glucose) and meanwhile repressed the stress response signaling pathway mediated by Hog1 MAPK. A working model, adopted and modified from b-locus regulatory network proposed in *U. maydis* [11], is depicted in Fig. 4 .

## Discussion

Investigation on molecular mechanism on *S. scitamineum* mating and/pathogenicity was impeded due to lack of *S. scitamineum* genome sequence, until 2014, when Que et al., published the first genome sequence of the pathogen [2]. More recently, a Brazil group published a complete genome assembly of *S. scitamineum*, as well as the fungal transcriptome profiles revealing the candidate genes unique to interaction with sugarcane [12]. Such genomic and transcriptome analyses have provided enormous convenience for functional study of mating and pathogenic genes in *S. scitamineum*. In current study, we conducted transcriptome analysis and comparison between mating vs non-mating haploid/mixture, which present useful information on the *b*-regulated gene expression cascade during *S. scitamineum* mating and/or filamentous growth. Our transcriptome analysis predicted 7341 unigenes (transcripts), which is similar to the predicted genome sizes of the three published *S. scitamineum* strains (http://www.ncbi.nlm.nih.gov/assembly/organism/49012/all/; Table 1). The GC-content of our identified unigenes is ranged from 50 to 60 %, peaking at 55 % (Fig. 1b), which is also consistent with the GC-content of these three genome projects (54.9, 54.8 and 55.04 % respectively). These data suggest that our *de novo* assembly of transcripts in *S. scitamineum* is valid for the identification of DEGs as well as GO and KEGG enrichment.

Our transcriptome analyses identified 357 DEGs in *SsΔMAT-1b* mutant compared to the wild-type *MAT-1*, and 391 DEGs in non-mating (*SsΔMAT-1b* X *MAT-2*) mixture compared to mating (*MAT-1* X *MAT-2*) mixture. Among them, 28 annotated genes (12 up-regulated and 16 down-regulated, Table 3) were common in these two sets of comparisons, thus are most likely associated with mating/filamentous growth and subject to regulation by bE/bW heterodimeric transcription factor.

In the enriched KEGG pathway, we noticed that sucrose/starch metabolism pathway was altered in the *SsΔMAT-1b* mutant in a way that intracellular glucose is predicted to be reduced and 1,3 β-glucan elevated. Also, glucoamylase encoded gene was identified as potentially b-locus induced (Table 3). Our results (Fig. 3b) showed in contrast to our prediction, that elevated glucose level repressed, but not promoted, filamentous growth and/or mating. We infer that the timing (24 h post mating) for detecting glucoamylase transcription might not be suitable, when at this time point the transcripts started translating into proteins. Therefore, the apparent low level of glucoamylase in non-mating sets would reflect active glucose production, and b-locus may actually repress glucoamylase during mating and/or filamentous growth. We further hypothesize that glucose may be channeled to synthesis of 1,3-β-glucan during *S. scitamineum* filamentous growth after mating and likely regulated by b-locus, through repression of glucoamylase. As 1,3-β-glucan is an anti-oxidant, its production may relief the cell from endogenous oxidative stress therefore Hog1 was not induced in wild-type condition. In b-deletion condition, glucose level may elevated and therefore repress filamentous growth; meanwhile the reduced1,3-β-glucan level resulted in endogenous oxidative stress and induction of Hog1 as a response. *SsΔMAT-1b* mutant was slightly more resistant to H2O2, likely due to hyper-induced Hog1. Our hypothesis was supported by the observation that glucose-depleted medium (SC) promoted filamentous growth in the mating mixture of *MAT-1* X *MAT-2* spores (Fig. 3c). It has been reported that glucose plays an important role in asexual/sexual sporulation in other pathogenic/filamentous fungi, including *Magnaporthe oryzae* [35], *U. maydis* [36], and *Fusarium graminearum* [37]. Also, glucose was reported to suppress mating competency in *Candida albicans* [16]. Our results fit well with the established notion that glucose promotes unicellular spore/cell production while represses filamentous growth, thus acting as a switch between dimorphic transition.

Another interesting observation from common DEGs and KEGG enrichment is that the stress-activating MAPK signaling pathway mediated by Hog1 was significantly up-regulated, in both *SsΔMAT-1b* mutant and *SsΔMAT-1b* X *MAT-2* mixture. One possibility is that, elevated glucose production in *SsΔMAT-1b* haploid resulted in reduced production of 1, 3-β-glucan, which is also known as an anti-oxidant. As a result, *HOG1* was transcriptionally induced in response to elevated intracellular oxidative level. Alternatively, *HOG1* may be repressed by the bE/bW transcriptional complex, directly or indirectly, during mating. Our tests showed that *SsΔMAT-1b* is less sensitive to oxidative stress. Meanwhile, repression on colonial growth caused by anti-oxidant GSH was more prominent with presence of glucose. Overall, these results suggest that Hog1 MAPK signaling may be repressed by the bE/bW transcriptional complex. Such observation is consistent with the reported function of the Hog1 ortholog in *Candida albicans* that negatively regulates its mating capacity [16]. However, we are not aware of whether *SsHOG1* is one of the direct targets (class I) genes of the bE/bW transcriptional complex, or among the indirect (class II) targets, as no obvious *b*-locus binding site (bbs [38, 39]) was predicted in the promoter region of *SsHOG1*.

It has been reported in *U. maydis* that GO categories “Cell Cycle”, “Chromosome” and “DNA metabolic process” were significantly enriched as b-down-regulated genes [40]. However, we observed that “DNA replication” was enriched as up-regulated GO terms in non-mating mixture (Fig. 2; Additional file 5: Table S5; GO: 0006260), and mitosis regulator Cek1 [24] and GAP Bem2 that related to mitosis [29] were up-regulated in non-mating sets, which may also account for the failure of mating, with deletion of *b*-locus in *MAT-1*.

## Conclusions

Overall, our transcriptome analysis contributes to prediction of candidate genes of the regulatory cascade of *S. scitamineum b*-locus, in terms of mating and/or filamentous growth after recognition of opposite sex mediated by the *a*-locus. In future, further investigation on such candidate genes would help elucidate molecular mechanism of *S. scitamineum* mating, including but not limited to, *b*-locus regulating cell fate decision, morphogenesis, carbon/nitrogen metabolism, mitosis, stress (oxidative) response, etc. This would certainly enrich our knowledge in fungal sexual differentiation and/or pathogenesis, and likely of great potential towards development/design of anti-fungal pathogen strategy.

### Ethics and consent to participate

Not applicable.

### Consent to publish

Not applicable.

### Availability of data and materials

All the data supporting our findings is contained within the manuscript and supplementary files.

## Additional files

Additional file 1: Table S1. List of All-Unigene *De novo* assembled in this study. (XLS 2360 kb) Additional file 2: Table S2. List of DEGs (*p* ≤ 0.05) in the *SsΔMAT-1b* mutant compared to the wild-type *MAT-1*. (XLS 396 kb) Additional file 3: Table S3. List of DEGs (*p* ≤ 0.05) in non-mating mixture of *SsΔMAT-1b* X *MAT-2*, compared to the mating mixture of *MAT-1* X *MAT-2 (XLS 185 kb)*
Additional file 4: Table S4. List enriched GO in *SsΔMAT-1b* mutant compared to the wild-type *MAT-1*. (XLS 41 kb) Additional file 5: Table S5. List enriched GO in non-mating mixture of *SsΔMAT-1b* X *MAT-2*, compared to the mating mixture of *MAT-1* X *MAT-2 (XLS 42 kb)*
Additional file 6: Table S6. List of enriched KEGG terms in *SsΔMAT-1b* mutant compared to the wild-type *MAT-1*. (XLS 47 kb) Additional file 7: Table S7. List of enriched KEGG terms in non-mating mixture of *SsΔMAT-1b* X *MAT-2*, compared to the mating mixture of *MAT-1* X *MAT-2*. (XLS 48 kb) Additional file 8: Figure S1. Starch/sucrose metabolism pathway (ko00500) common in both haploid and mating sets. Red box denotes up-regulated genes, and green box are down-regulated genes. The source of image is from KEGG pathway database (http://www.kegg.jp/) developed by Kanehisa Laboratories, and is allowed to reproduced for academic purpose. (TIF 1572 kb) Additional file 9: Figure S2. MAPK signaling pathway (ko04010) common in both haploid and mating sets. Red box denotes up-regulated genes, and green box are down-regulated genes. The source of image is from KEGG pathway database (http://www.kegg.jp/), developed by Kanehisa Laboratories, and is allowed to reproduced for academic purpose. (TIF 1373 kb)
