# Supplementary figures and images for: Transcriptome analysis of Sporisorium scitamineum reveals critical environmental signals for fungal sexual mating and filamentous growth
Source: BMC Genomics. 2016 May 16;17:354. doi: 10.1186/s12864-016-2691-5 (PMC4867532; doi:10.1186/s12864-016-2691-5)

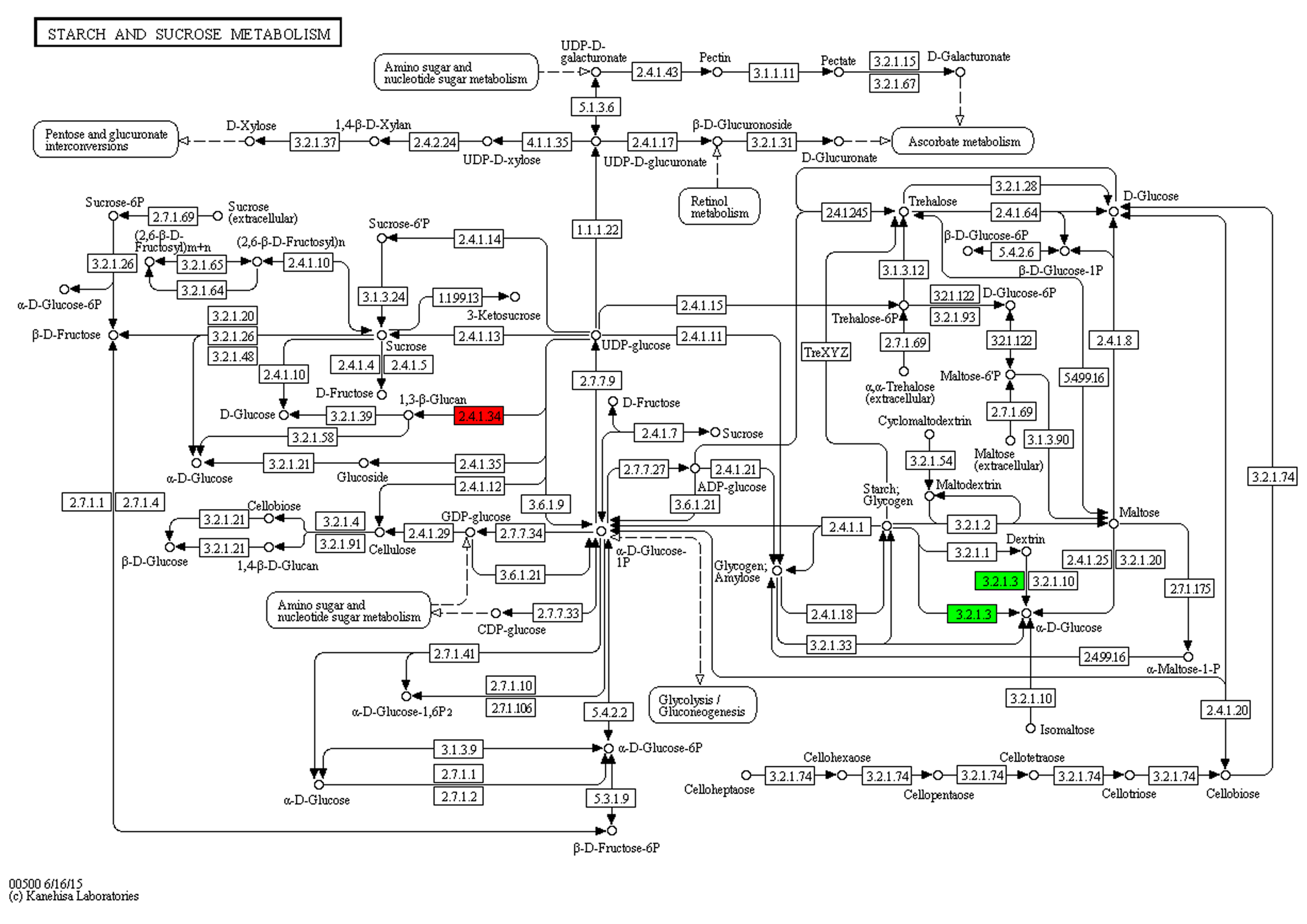

Supplement: Additional file 8: Figure S1. — Starch/sucrose metabolism pathway (ko00500) common in both haploid and mating sets. Red box denotes up-regulated genes, and green box are down-regulated genes. The source of image is from KEGG pathway database (http://www.kegg.jp/) developed by Kanehisa Laboratories, and is allowed to reproduced for academic purpose. (TIF 1572 kb) [file 12864_2016_2691_MOESM8_ESM.tif]

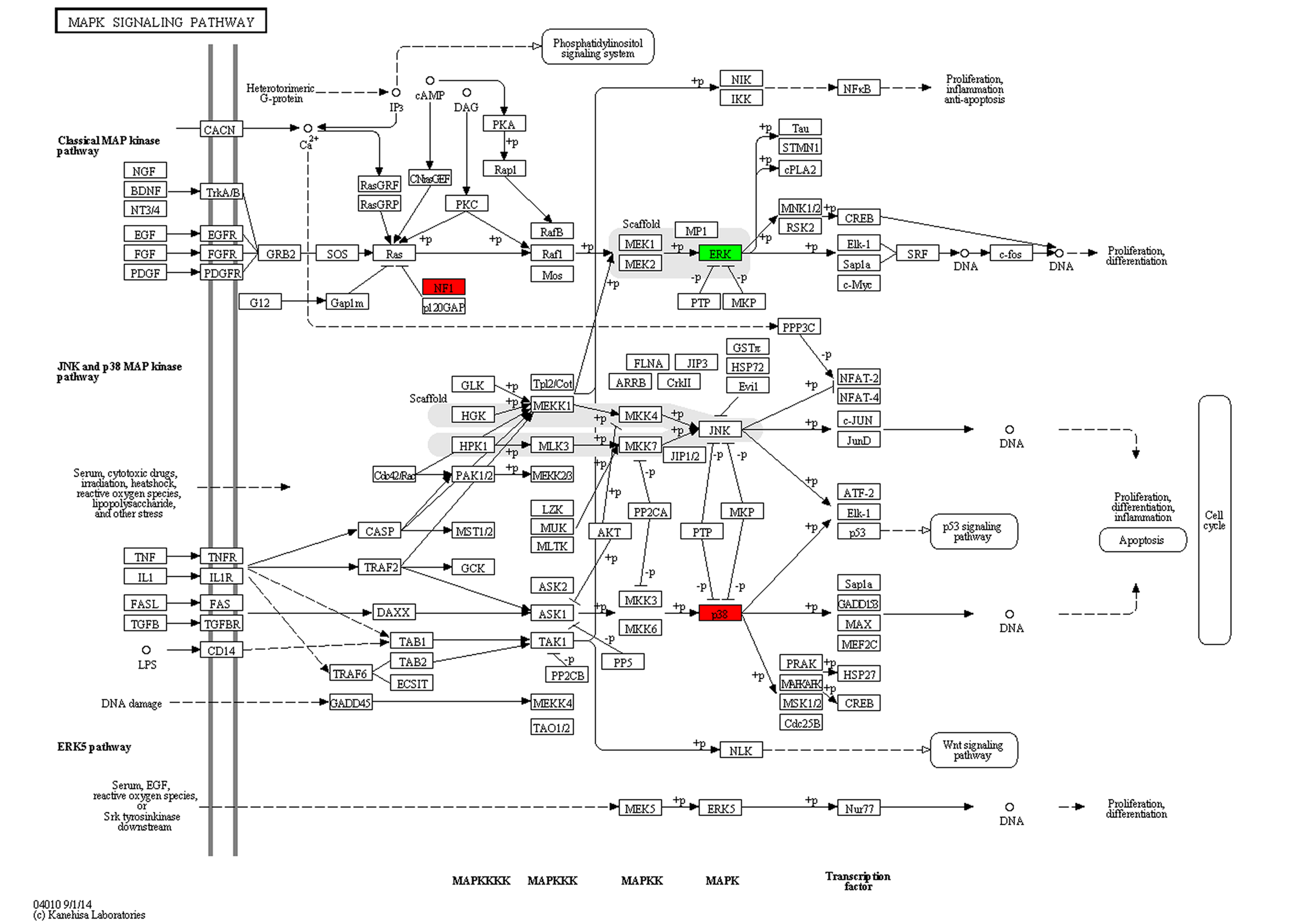

Supplement: Additional file 9: Figure S2. — MAPK signaling pathway (ko04010) common in both haploid and mating sets. Red box denotes up-regulated genes, and green box are down-regulated genes. The source of image is from KEGG pathway database (http://www.kegg.jp/), developed by Kanehisa Laboratories, and is allowed to reproduced for academic purpose. (TIF 1373 kb) [file 12864_2016_2691_MOESM9_ESM.tif]
